# Acknowledgement to Reviewers of *JPM* in 2019

**DOI:** 10.3390/jpm10010001

**Published:** 2020-01-04

**Authors:** JPM Editorial Office

**Affiliations:** MDPI, St. Alban-Anlage 66, 4052 Basel, Switzerland

The editorial team greatly appreciates the reviewers who have dedicated their considerable time and expertise to the journal’s rigorous editorial process over the past 12 months, regardless of whether the papers are finally published or not. In 2019, a total of 52 papers were published in the journal, with a median time to first decision of 24 days and a median time from submission to publication of 45 days. The editors would like to express their sincere gratitude to the following reviewers for their generous contribution in 2019:
Andrews, LesleyLeo, Carlo GiacomoAntipova, VeronicaLi, YvonneBaldovino, SimoneLi, YanBandres-Ciga, SaraLikic, RobertBaranova, AnchaLokshin, AnnaBatko, BogdanMalpeli, GiorgioBodurtha, JoannMartin, CesarBoisvert, Francois-MichelMason-Suares, HeatherBolanowski, MarekMatzeu, GiusyBraakhuis, AndreaMelo, Sonia A.Brown, Jacob T.Meng, JinhongCapobianco, EnricoMolyneaux, PhilipCaprini, JosephMorten, BriannaCarmi, ShaiMoyle, LouiseChallagundla, KishoreMurray, Michael F.Chmurzynska, AgataParker, Elizabeth A.Čipak Gašparović, AnaPatel, Jai N.Clementi, EmilioPergament, EugeneCohen, AdamPettinato, GiuseppeColeman, JonathanPhillips, JacquelineCordeiro, Ana SaraPiro, GenyCrona, DanielPrimig, MichaelDe Berardis, DomenicoPulliero, AlessandraDebeljak, NatašaPuri, KritiDeWald, TracyRadtke, Jan P.Dijkstra, JoukeReponen, JarmoDocea, AncaRoss, LainieDorfman, RuslanSaini, SharanjotDuconge, JorgeSandman, LarsEarl, JulieSchisler, JonathanEngvall, JanSchmid, GiovanniEwing, AdamScott, Erick R.Ferguson, BradleySeeboeck, RitaGauthier, PascaleSenter, LeighaGhoshal-Gupta, SampaSharfaei, SadafGlueck, Charles J.Sharma, NaveenGolemi, IvaSmith, Judith A.Gouin-Thibault, IsabelleSquassina, AlessioGrzybowska, EwaStewart, AlexandreHaboubi, NadimSujobert, PierreHart, M. RaganTougeron, DavidHowes, Laurence GuyVafiadaki, ElizabethHvas, Anne-MetteValentine, Rudy J.Indiveri, CesareVan Putten, Maaike VanIsidoro-García, MaríaVimaleswaran, Karani S.Janssen, AnnaWang, KaiJennings, Lawrence J.Williams, JanetJerry, D. JosephWinter, AlexanderJose, JineyWu, Alan H.Juth, NiklasZaiou, MohamedKim, Raymond H.Zaiss, DietmarKiuchi, Márcio GalindoŻebrowska, AleksandraKlovins, JanisZhou, JingyingKulak, WojciechZhu, YuLara-Otero, KarlenaZiegelstein, Roy

